# COVID-19 infection as a new risk factor for penile Mondor disease

**DOI:** 10.1186/s12894-022-01002-x

**Published:** 2022-04-12

**Authors:** Krzysztof Balawender, Anna Pliszka, Agata Surowiec, Sebastian Rajda

**Affiliations:** 1 Clinical Department of Urology and Urological Oncology, Municipal Hospital in Rzeszow, Rycerska 4, 35-241 Rzeszow, Poland; 2 Morphological Sciences Department, Institute of Medical Sciences, Medical College of Rzeszow University, Leszka Czarnego 4, 35-301 Rzeszow, Poland

**Keywords:** COVID 19 virus infection, Thrombophlebitis, Penile diseases

## Abstract

**Background:**

Penile Mondor disease is a superficial dorsal vein thrombophlebitis of the penis, which mainly affects young and middle-aged men. It generally manifests as a visible painful cord located along the dorsal surface of the penis with signs of skin inflammation. The condition is usually self-limiting, but in severe cases a surgical procedure may be necessary in addition to pharmacological treatment. Coronavirus disease 2019 (COVID-19) caused by SARS-CoV-2 is associated with a frequent incidence of thrombophilia; therefore, such a prothrombotic state during infection may be a significant risk factor for penile Mondor disease.

**Case presentation:**

The 34-year-old patient reported moderate pain felt on the surface of the penis. During the medical interview, the patient did not admit significant risk factors for Mondor Disease, apart from the previous, a month earlier COVID-19 disease. Examination revealed swelling erythema and a thick indurated cord on the surface of the penis. Color Doppler ultrasound was performed to confirm assumptions and exclude thrombosis of other penile vessels. Based on visible clots in the course of the superficial penile vein and after exclusion of vasculitis due to autoimmune disease the diagnosis of penile Mondor disease was made. Pharmacological therapy was implemented to further break down the clot and prevent rethrombosis in the penile vessels. The patient did not report any treatment complications and returned for a control visit, which revealed complete clot dissolution on ultrasound; therefore, complete recovery was stated.

**Conclusions:**

This case report presents the correlation between SARS-Cov-2 infection and penile Mondor disease, based on the confirmed influence of COVID-19 on the pathophysiology of thrombosis. It can be concluded that COVID- 19 is a risk factor for Mondor disease, as in the presented case the virus was the only prothrombotic risk factor for the patient. Consequently, the possibility of developing thrombosis in the form of penile Mondor disease should be taken into account among patients with post-COVID-19 and active SARS-Cov-2 infection.

## Background

Mondor’s disease is a superficial vein thrombosis. It was first described in 1939 by Henri Mondor, who found a syndrome of superficial sclerosing thrombophlebitis of the veins of the anterior thoracic wall [1]. In 1955 Braun Falco described a generalized form of penis disease, and 3 years later Helm and Hodge reported isolated thrombosis of the dorsal superficial vein of the penis (penile Mondor disease) [2, 3]. Penile Mondor disease (PMD) is a rare disease with a 1.39% incidence rate in the group of young men (20–40 years old) [4]. The disease is probably underreported and underdiagnosed due to the fear and animosity of patients to consult a doctor. The typical PMD patient profile is a 21- to 70-year-old sexually active man [5]. The exact cause of PMD is unknown. However, there are some conditions that might lead to PDM. It includes a hypercoagulative state, vasculitis, sexually transmitted diseases, prolonged sexual activity (sexual intercourse or masturbation), trauma/surgical intervention to the pelvis or external genitalia, and tumors located in the pelvis [6, 7]. The first case of COVID-19 was reported in December 2019 in Wuhan, China, and spread rapidly throughout the world, causing more than 5 million deaths [8]. The symptoms manifested by the patients are very diverse, ranging from minimal to severe. The opacification of the ground glass pulmonary seen on computed tomography is characteristic [9]. Although the most common manifestation of the severe form of the disease is related to the respiratory tract, other non-respiratory effects, including long-lasting, have been described [10, 11]. Patients with COVID-19, especially with severe infection, have a greater spread of thrombosis [12]. The available data suggest that 21–69% of critically ill patients will develop thromboembolism [13]. Most of them show some signs of hypercoagulability: increased fibrinogen, high circulating D-dimer concentrations, elevated PT and APTT [14]. The pathomechanism of thrombophilia induced by SARS-CoV2 remains unclear. It is probably associated with direct endothelial damage and inflammation factors such as cytokines and reactive oxygen species [15]. Although the most common thrombotic state of COVID-19 infection is deep vein thrombosis and pulmonary embolism, so far published isolated reports indicate that it can also manifest itself as thrombosis of the penis vessels [16–18]. Furthermore, a report shows that past COVID-19 penile vessel thrombosis can appear not only as PMD, superficial thrombosis of the dorsal penile vein, but also as deep thrombosis of the dorsal penile vein or circumflex vein [17, 19].

The purpose of this article is to focus particular attention on a possible correlation between SARS-Cov-2 infection and PMD. This case report shows that PMD can appear without any previous thrombotic history, vasculitis, sexually transmitted diseases, denying recent vigorous sexual activity or penis trauma, it would have seemed completely healthy man, who had only had a mild SARS-Cov-2 infection previously, which indicates SARS-Cov-2 infection as a possible risk factor for PMD. The prothrombotic state during COVID-19 infection can lead to thrombosis episodes even months after complete recovery and even during mild infection [20, 21].

## Case presentation

A 34-year-old male patient, university mathematics lecturer, with BMI 21,5 was admitted to the emergency department due to pain located on the surface of the penis that had been ongoing for the last 3–4 days. He did not report fever, infection symptoms, dysuria, or pain in other localization. He denied prior vigorous sexual intercourse last week and took medications from the phosphodiesterase-5 enzyme inhibitors group, prothrombotic drugs, or any other drugs. He classified his smoking status as never-smoker and declined allergies or autoimmune diseases such as Buerger's disease, Behçet's disease, lupus erythematosus, and polyarteritis nodosa. When asked about recent infections, the patient said that a month ago he recovered from an outpatient treated 7-day lasting COVID-19 infection confirmed by the PCR test. He suffered from sore throat, headache located around the frontal and maxillary sinuses, runny nose, cough, general weakness, and denied symptoms such as fever, dyspnea, taste, or smell loss. The disease developed without serious complications; therefore, it can be classified as mild. The patient did not notice any previous episodes of thrombosis in his life. The history of thrombotic, oncological, and autoimmune diseases in the family was negative.

**Fig. 1:**
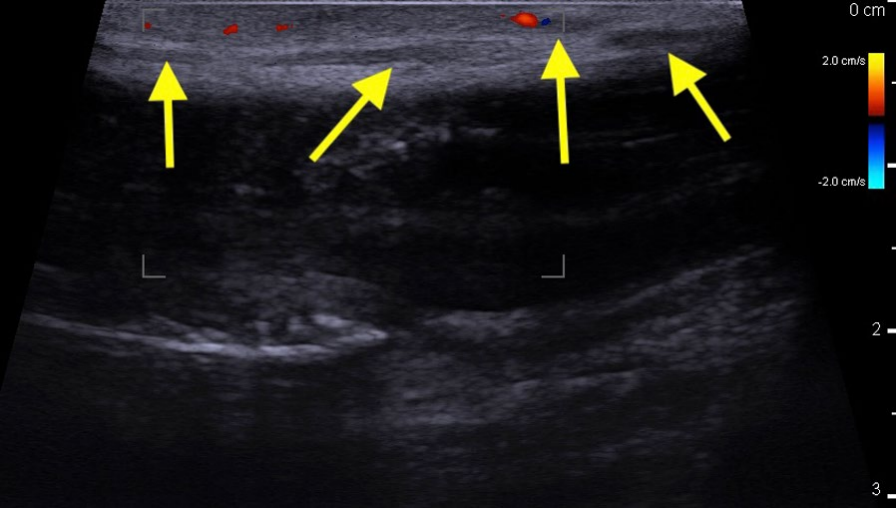
Doppler ultrasound of penis: longitudinal section. Yellow Arrow—Dorsal Superficial penile vein without flow

**Fig. 2:**
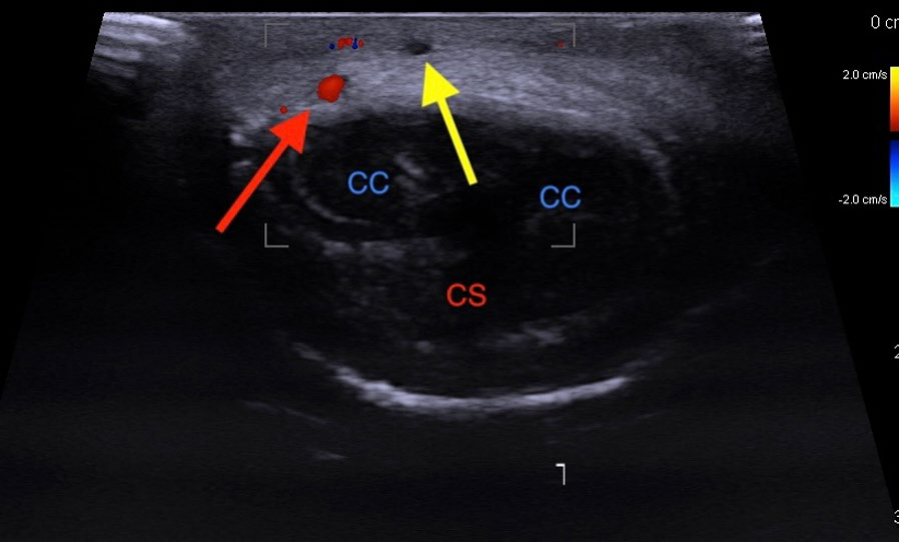
Doppler ultrasound of penis: cross section, CC—Corpus Cavernosum Penis. CS—Corpus Spongiosum Penis, Yellow Arrow—Dorsal Superficial penile vein without flow, Red Arrow—Right Dorsal penile artery

Examination revealed a thick, thick cord that extends from the base to the corona in the course of the superficial dorsal penile vein with signs of skin inflammation, such as a slight swelling erythema. Ultrasound of the penis was performed. It revealed that there was no flow through the vessel in the middle of the superficial dorsal penile vein (Figs. 1,2). Furthermore, the deep dorsal penile vein was invisible without flow in both sections, probably due to the vessel clot above compression. The results of the laboratory tests did not reveal any significant signs of prothrombotic state (Table 1).

**Table 1:** Patient’s laboratory tests results

Test	Patient result	Result [normal value]
Platelet’s count	290,000	[150,000–400,000/μl]
White Blood cells count	6500	[4000–10,000/μl]
Partial Thromboplastin Time (PTT)	33	[30–40 s]
Prothrombin Time (PT)	11 s	[13 s]
International Normalized Ratio (INR)	1.1	[0.9–1.2]
Ferritin		[15–400 µg/l]
D-dimer	480	[up to 500 µg/l]
Fibrinogen	375 mg/dl	[200–400 mg/dl]
Anti-thrombin III	96%	[80–120%]
Protein C	70%	[65–145%]
Protein S	86%	[63.5–167.9%]
Lupus Anti-coagulant	27 MPL/ml	≤ 40 s
Anti-cardiolipin (IgM)	4 MPL/ml	< 12 MPL/ml
Anti-cardiolipin (IgG)	3 MPL/ml	
Anti-phospholipid (IgM)	3 MPL/ml	
Anti-phospholipid (IgM)	6 MPL/ml	

Due to the absence of other symptoms or diagnosis of autoimmune diseases in the patient's life, a negative family history, and no evidence of the presence of antibodies for autoimmune diseases in laboratory tests, Buerger's disease, Behçet's disease, lupus erythematosus, and polyarteritis nodosa were ruled out. In this case, the previous COVID-19 was only a risk factor and could substantially lead to PMD. Knowledge of the topography of the penis vessels by ultrasound allowed us to differentiate the location of the thrombosis in the superficial dorsal penile vein from the thrombosis in the deep superficial penile vein that had been compressed by clots in the vessel above. Based on clinical symptoms, possible risk factors such as SARS-Cov-2 infection, and performed lab tests and ultrasound, the PMD diagnosis was made.

Pharmacological treatment was implemented. The patient received a therapeutic dose of high molecular weight heparin (enoxaparin 1 mg per kilogram body weight twice daily subcutaneously) and analgesics (100 mg diclofenac p.o.) for pain relief. Additionally, 20 g of heparin in cream was applied to the surface of the penis along the widened vein. After 2 days, swelling decreased and the superficial dorsal penile vein area softened, indicating the beginning of clot dissolution and the start of recovery. The patient was sent home with appropriate recommendations, such as continued use of high molecular weight heparin, heparin cream, and analgesics for 4 weeks, preventive instruction to stop sexual activity until symptoms disappeared and to return to the clinic immediately if symptoms worsen or do not improve within the next few days. The control visit was planned in 4 weeks. The patient did not report any complications. He came for a control visit, during which a Doppler ultrasound was performed. It revealed almost complete disintegration of the clots and correct flow through both the superficial and deep penile veins. Flow through other vessels was also not hampered.

## Discussion and conclusions

The Virchow triad determines three necessary factors that lead to thrombus formation, which are hypercoagulability, endothelial dysfunction, and hemodynamic changes such as turbulent flow caused by vessel stasis. The COVID-19 virus leads to endothelial dysfunction. The primary genesis of this phenomenon is the release of inflammatory cytokines (TNF-α, IL-6, IL-1β) release in response to the entry of SARS-CoV-2 into the body to promote a hyper-inflammation state that also contains vessel inflammation, which can manifest itself selectively in different parts of the body where it occurs with or without participation of other external factors. This phenomenon contributes to damage to the endothelium of vessels, where the tiniest blood vessels are the most susceptible to injury due to their fragility. Inflamed endothelial cells release excessively von Willebrand factor multimers, the prothrombotic glycoprotein responsible for the aggregation and adhesion of platelets to wounded tissue. As a proof of the contribution of SARS-CoV-2 to the inflammatory prothrombotic state, it is a fact that the levels of von Willebrand factor multimers analyzed were significantly elevated among patients with COVID-19 [22]. Moreover, directly produced inflammatory cytokines induce genes responsible for other mediators like reactive oxygen species prostanoids, nitric oxide, leukotrienes, bradykinin, platelet activating factor causing further endothelium damage and additionally sparking this vicious cycle of vessel integrity damage. Platelet activating factor overproduction leads to easier thrombin generation and fibrin formation, which even more so with large amounts of von Willebrand factor multimers release leads to blood hypercoagulability and creates susceptibility to thrombus formation [23–26]. The dysfunctional endothelium is unable to produce NO, causing its deficiency, which is responsible for the disturbance of the vasorelaxation effect. Hemodynamic changes were observed among patients with COVID-19 as vessels vasoconstriction [27]. Therefore, three elements of the Virchow triad occur during SARS-Cov-2 infection, where there is usually a correlation with the severity of the disease, respectively. After all, we can state that COVID-19 is a highly prothrombotic disease, and, moreover, the patient’s response is not always predictable, making it even more dangerous, which we cannot underestimate.

Study of 30 men with PMD shows that among 4 of them previous infection was the cause of the disease [28, 29]. Additionally, there are multicentric studies which reports strong correlation between COVID-19 and prothrombotic state development, which is a proved risk factor for PMD, although further research about direct impact of COVID-19 on PMD should be done [30–33].

PMD can be revealed by the patient's medical history and physical examination, which usually shows a thrombosed vein along the penis or on the dorsal surface [19, 28, 34]. The most known clinical presentation is painful, tender, subcutaneous induration. Usually, palpation is accompanied by increased pain [35]. Doppler ultrasound of the dorsal vein is required to confirm the disease and perform a reliable differential diagnosis. Doppler ultrasound reveals the extension and area of vein thrombosis. No more radical diagnostic methods are required. However, when some etiopathogenetic causes are suspected, such as trauma, cancer, or thrombophilia, other invasive studies may be necessary [28, 35].

Obtaining information about risk factors such as previous, even mild, SARS-Cov-2 infection from the patient will speed up the diagnosis process and minimize the severity of the disease. When it comes to PMD, it is crucial to make a correct diagnosis and implement proper treatment as soon as possible to avoid serious thrombotic complications [36, 37].

PMD is usually a self-limiting disease that resolves in 4 to 6 weeks. Usually, a conservative approach is the best option for treatment. Topical anticoagulants or non-steroidal inflammatory agents, taken orally, are generally used to reduce pain and inflammation. However, there is no strong position in the literature to use anticoagulation. [38, 39]. It is recommended to abstain from sexual activity for about 6 weeks until symptoms resolve [40]. When the patient does not improve during 4–6 weeks of conservative treatment, surgery is required. It consists of thrombectomy or superficial thrombosed dorsal vein resection [41–43].

Our case management included a thoroughly conducted medical interview, during which the patient did not admit significant risk factors for PMD. After interview and examination, color Doppler ultrasound was performed to confirm assumptions and exclude thrombosis of other penile vessels, therefore there was no need to expand the diagnostic process with more radical methods. The decision about therapeutic intervention as pharmacological treatment was made based on the generally accepted procedure rules for PMD and the low severity of the disease. Due to the visible improvement after a few hours, more invasive treatment such as surgery was unnecessary. Complete recovery was declared after 1 month of outpatient therapy.

This case report shows that although PMD is a rather rare condition, clinicians should be aware of its occurrence as a complication of COVID-19. Moreover, the article presents a possible significant correlation between SARS-Cov-2 infection and PMD due to the fact that COVID-19 was one and only possible thrombosis risk factor in our patient; therefore, we can confirm the thesis of SARS-Cov-2 infection as a PMD risk factor. This article emphasizes the role of suspecting thrombosis among patients with paradoxically mild COVID-19 even months after recovery.

## Data Availability

Not applicable. All data (of the patient) generated during this study are included in this published article and its supplementary information files.

## Abbreviations

PMD: Penile Mondor disease
COVID-19: Coronavirus Disease 2019
